# Anti-Angiogenic and Anti-Metastatic Activity of Synthetic Phosphoethanolamine

**DOI:** 10.1371/journal.pone.0057937

**Published:** 2013-03-14

**Authors:** Adilson Kleber Ferreira, Vanessa Morais Freitas, Débora Levy, Jorge Luiz Mária Ruiz, Sergio Paulo Bydlowski, Rose Eli Grassi Rici, Otaviano Mendonça R. Filho, Gilberto Orivaldo Chierice, Durvanei Augusto Maria

**Affiliations:** 1 Biochemistry and Biophysical Laboratory, Butantan Institute, Sao Paulo, Brazil; 2 Experimental Physiopathology, Faculty of Medicine, University of Sao Paulo, Sao Paulo, Brazil; 3 Department of Cell and Developmental Biology, University of Sao Paulo, Sao Paulo, Brazil; 4 Laboratory of Genetics and Molecular Hematology (LIM-31), Faculty of Medicine, University of Sao Paulo, Sao Paulo, Brazil; 5 Department of Chemistry and Polymers Technology, University of Sao Paulo, Sao Carlos, Brazil; 6 University of Uberaba, Minas Gerais, Brazil; 7 Department of Surgery, Faculty of the Veterinary Medicine and Zootecny, University of Sao Paulo, Sao Paulo, Brazil; Children’s Hospital Boston & Harvard Medical School, United States of America

## Abstract

**Background:**

Renal cell carcinoma (RCC) is the most common type of kidney cancer, and represents the third most common urological malignancy. Despite the advent of targeted therapies for RCC and the improvement of the lifespan of patients, its cost-effectiveness restricted the therapeutic efficacy. In a recent report, we showed that synthetic phosphoethanolamine (Pho-s) has a broad antitumor activity on a variety of tumor cells and showed potent inhibitor effects on tumor progress *in vivo*.

**Methodology/Principal Findings:**

We show that murine renal carcinoma (Renca) is more sensitive to Pho-s when compared to normal immortalized rat proximal tubule cells (IRPTC) and human umbilical vein endothelial cells (HUVEC). *In vitro* anti-angiogenic activity assays show that Pho-s inhibits endothelial cell proliferation, migration and tube formation. In addition, Pho-s has anti-proliferative effects on HUVEC by inducing a cell cycle arrest at the G2/M phase. It causes a decrease in cyclin D1 mRNA, VEGFR1 gene transcription and VEGFR1 receptor expression. Pho-s also induces nuclear fragmentation and affects the organization of the cytoskeleton through the disruption of actin filaments. Additionally, Pho-s induces apoptosis through the mitochondrial pathway. The putative therapeutic potential of Pho-s was validated in a renal carcinoma model, on which our remarkable *in vivo* results show that Pho-s potentially inhibits lung metastasis in *nude* mice, with a superior efficacy when compared to Sunitinib.

**Conclusions/Significance:**

Taken together, our findings provide evidence that Pho-s is a compound that potently inhibits lung metastasis, suggesting that it is a promising novel candidate drug for future developments.

## Introduction

Every year, around 208,500 new cases of kidney cancer are diagnosed worldwide. Among them, the renal cell carcinoma (RCC) represents the third most common urological malignancy [1]. It is a rare disease that accounts for about 2–3% of all solid tumors in adults and represents about 85% of all kidney cancers. It arises from the renal epithelium and although its etiology is not known, approximately 4% of the RCCs are present in the complex of hereditary syndromes [2].

The increase in oxidative stress has been extensively investigated as a potential inducer of cancer and of malignant progression [3]. The microenvironment and stromal components are directly responsible for the enhancement of tumor induction and progression caused by oxidative stress [4]. Reactive oxygen species (ROS) act as modulators of cellular signaling, inducing tumor proliferation and contributing to angiogenesis and metastasis [5]. In a recent report a change in the redox status was observed during tumor growth in the tumor tissue of patients with RCC. In contrast, ROS and NO did not increase in patients with benign tumors when compared to patients with malignant tumors. In patients with metastatic disease who had their tumor surgically removed, ROS production did not decrease and it was associated with the residual disease [6].

The von Hippel–Lindau (VHL) tumor suppressor gene located on chromosome 3p25 has a high penetrance and confers a predisposition for the development of highly vascularized tumors [7]. This gene encodes the VHL protein that prevents the proteolysis of HIFα subunits. It is a regulator of the hypoxic stress response and its up-regulation genes that encode the vascular endothelial growth factor (VEGF). The main strategy in the RCC treatment is the inhibition of angiogenesis by VEGF signaling [8]. The cellular effects of VEGF are mediated through receptor tyrosine kinase VEGFR-1 (Flt-1) and VEGFR-2 (KDR/Flk-1) that are selectively expressed in endothelial cells [9]. Upon binding of VEGFR, the receptor promotes proliferation and migration of these cells [10]. Presently, the first generation of tyrosine kinase inhibitors, including Sunitinib, are the standard drugs in the treatment of RCC. However, the development of new compounds that block VEGF or VEGFR, with anti-angiogenic activity can be a future candidate for the treatment of RCC [11], [12].

The primary amine phosphoethanolamine is a precursor of phosphatidylcholine and phosphatidylethanolamine and is involved in the turnover of cell membranes’ phospholipids [13]. Both phospholipids take part in the lipid signaling pathways acting either as ligands or by generating intermediate substrates [14]. In a previous study, synthetic phosphoethanolamine (Pho-s), a central precursor in the biosynthesis of membrane phospholipids, showed a high antitumor activity in an *in vivo* melanoma model, reducing the tumor growth and the number of metastasis. The histological and histochemical analysis of the tumors showed that treatment with Pho-s reduces the size, number of vessels and neo-vascularization. Thus, it suggests that Pho-s show anti-angiogenic activity [15]. However, the molecular mechanism responsible for the anti-tumor properties of Pho-s is still under investigation. In the present work we have investigated the *in vitro* anti-proliferative and anti-angiogenic effects of Pho-s. In parallel we also evaluated its therapeutic effects in a metastatic model of the renal carcinoma.

## Materials and Methods

### Ethics Statement

All experimental procedures were carried out in accordance with the guidelines for animal experimentation determined by the Butantan Institute Animal Care committee. The study protocol was approved by the Butantan Institute for the Use of Animal (process number 566/09).

### Cell Culture

Carcinoma renal murine (Renca) [16] and immortalized rat proximal tubule cells (IRPTC) [17] were kindly provided by Dr. Maria Helena Bellini (Institute of Energy and Nuclear Research, IPEN, São Paulo, Brazil) and Dr. Maria Oliveira de Souza (Department of Physiology and Biophysics, Institute of Biomedical Sciences, University of São Paulo, São Paulo, Brazil), respectively. Human umbilical vein endothelial cells (HUVEC -CRL 1730) were obtained from the American Type Culture Collection (Mannasa, VA, USA). All cell lines were cultured in RPMI- 1640 medium, supplemented with 10% fetal bovine serum, 100 units/ml penicillin, 100 mg/ml streptomycin and at 37°C in a humidified atmosphere with 5% CO_2_.

### MTT Colorimetric Assay

Renca, IRPTC and HUVEC cells were plated in 96-well flat bottom tissue culture plates at a concentration of 1×10^4^ cells/100 µl/well. The cells were allowed to grow for 24 h, and treated with concentrations of Pho-s ranging from 10 mM to 100 mM and Sunitinib (positive control) from 0.01 µM to 100 µM. To evaluate the cell proliferation on Huvecs, cells were treated with Pho-s ranging from 2.5 mM to 15 mM and Sunitinib from 1 µM to 5 µM. After 24 h of treatment, cell viability was determined by MTT (3-[4,5 dimethylthiazol-2-yl]-2,5 diphenyltetrazolium bromide). Briefly, 20 µl of MTT reagent (Sigma-Aldrich, St. Louis, USA) were added to each well at a final concentration of 5 mg/ml, incubated for 4 h at 37°C and centrifuged at 2000 rpm for10 min. The medium was discarded and 100 µl of dimethyl sulfoxide were added to each well. Each experiment was performed using six replicates for each drug concentration and was repeated in triplicate.

### Cell Cycle Analysis and Cell Proliferation Assay

HUVEC were synchronized by deprivation of serum for 24 h and induced to reenter the cell cycle by the subsequent addition of serum. Then, cells were treated for 24 h with 90 mM Pho-s or 1 µM Sunitinib. Staurosporine (ST) was used as positive control at the concentration of 5 µM to induce sub-G1 peak as an indicative of apoptosis. Then, cells were collected and fixed with cold 70% ethanol and stored at −20°C. Cells were washed and resuspended in PBS and were incubated at 37°C for 45 min with10 mg/ml RNase and 1 mg/ml Propidium Iodide (SIGMA, St. Louis, MO), then incubated for 30 min at 37°C. For proliferation assay the HUCEC were incubated with carboxyfluorescein diacetate succinimidyl ester (CFDA-SE; Molecular Probes, Inc., Eugene, OR) (0.5 µM for 10 min at room temperature). After, cells were washed thrice with complete medium, and seeded at 10^6^ cells/ml. Following, cells were treated with 10 mM Pho-s or 1 µM Sunitinib for 96 h and analyzed by flow cytometry. The data were reported as “division index” from four independent experiments. Flow cytometric analysis was performed using a FACScan flow cytometry system (Becton Dickinson, San Jose, CA). The cell proliferation analysis was carried out using WinMDI Version 2.8. The percentage of cells in the different cell cycle phases was determined using Modfit LT software (Verity Software House, Topsham, ME).

### Tube Formation Assay

Matrigel matrix was added to each well of a chilled 24 well plate and allowed to solidify for 30 min at 37°C. A suspension of 5×10^4^ HUVEC in RPMI was added on to the matrigel (Trevigen, Gaithersburg, USA) coated wells and the cells were treated with 10 mM Pho-s or 1 µM Sunitinib. After incubation of 48 hr at 37°C, tube formation was observed using Olympus CK2 light microscope and the total tube length, total branching points and total loops was measured using the WimTube Key Metrics Program (Wimasis GmbH, Munich, Germany).

### Wound-healing Assay

HUVEC (2.5×10^5^ cells) were grown to confluence in a 12-well plate, placed in medium containing 1% serum for 24 h at 37°C in an atmosphere of 5% CO_2_. Upon reaching confluence, the cell layer was scratched with a sterile plastic tip and then washed twice with culture medium. Then, serum was increased to 5% to facilitate cell migration and the cells were treated with either 10 mM Pho-s, 1 µM Sunitinib, 10 µM Roscovitine (positive control), 10 µM BAY 11-7082 (positive control) or10 ng/ml VEGF for 48 h. HUVEC migration was recorded using a Nikon TE2000E microscope system (Nikon Instrument). The area of wound healing was calculated using WimScratch software.

### Real-time PCR

HUVEC were treated with 10 mM Pho-s or 1 µM Sunitinib for 12 h, PBS was used as control. Brief, RNA extraction from sub-confluent treated or non-treated cells was performed using 1.0 ml of Trizol (Invitrogen) for each Huvecs 1×10^6^ cells of sample according to manufacturer’s recommendation. RNA integrity was assayed by agarose gel electrophoresis and treated with DNAse (RQ1 RNAse free DNAse – Promega). cDNA and PCR were performed using SuperScript III Platinum one-step qRT-PCR Systems (Invitrogen). Gene expression was measured in 7500 Fast (Applied Biosystems) using GAPDH (Hs99999905_m1) as endogenous gene. Taqman gene expression assay from Applied Biosystems were performed for cyclin D1 and VEGFR1 using Hs00765553_m1 and Hs01052937_ml genes, respectively.

### Microscopy Confocal Analysis of Renca Cells Using FITC-phalloidin and PI

After incubation with 90 mM Pho-s for 6 h, the cells were fixed in 4% paraformaldehyde in PBS for 1 h. To analyze the effect of Pho-s in actin filaments, cells were labeled with FITC-phalloidin (Invitrogen-Molecular Probes, Eugene, OR) in a buffer containing 0.5% Triton X-100, 1 mg/ml RNase in 2× SSC. Nuclei were counterstained with propidium iodide (PI) (Invitrogen-Molecular Probes). The preparations were mounted on slides with Pro Long (Invitrogen-Molecular Probes). Fluorescence images were obtained in the Zeiss LSM 510 laser scanning confocal microscope (Carl Zeiss LSM 700; Leica, Mannheim, Germany). Post-acquisition image processing, background correction, adjustment of brightness and contrast and export to tiff format, were done with Image J software (version 14.1) National Institutes of Health (Bethesda, Maryland, USA).

### Apoptosis Detection Assay

For the detection and evaluation of apoptosis, the cells were plated in 6-well culture plates, grown overnight and pre-treated with 2.5 mM cyclosporin A (CsA), a specific inhibitor of cyclophilin and with 40 µM Z-VAD-fmk, a pan-caspase inhibitor, for 2 h, followed by the addition of 90 mM Pho-s. ST was used as a positive control at the concentration of 5 µM for apoptosis induction. The adherent and floating cells were collected and washed in PBS. The cells were then centrifuged and the cell pellet was suspended with binding buffer (100 µl). Next, the cells were stained with Annexin V-FITC/PI apoptosis detection kit (BD Bioscience), incubated for 15 min at room temperature in the dark. After incubation, 400 µl of binding buffer were added and cells were analyzed by flow cytometry (FACScalibur, Becton Dickinson) using CellQuest software, determining the percentage of apoptotic cells. A minimum of 10,000 events were acquired for each sample.

### Measurement of Mitochondrial Membrane Potential

Renca cells (10^6^ cells/well) were seeded in 6-well plates and incubated for 12 h, then treated with 90 mM Pho-s or 5 µM ST. After 12 h, the cells were harvested and washed once with PBS and stained with 10 mM JC-1 for 15 min at 37°C. Next, the cells were washed once with PBS and kept at 4°C during measurements. The mitochondrial membrane potential (ΔΨ) was measured by flow cytometry (FACScalibur, Becton Dickinson). A total of 10,000 cells/sample were analyzed and the mean fluorescence intensity recorded.

### Detection of Caspase-3 activity by Fluorometric Assay

The Renca cells were treated for 12 h with 90 mM Pho-s or 5 µM ST, then lysed in 100 ml of 1× lysis buffer. The lysates were collected and stored at –70°C until analyzed. Briefly, 50 ml of 1× reaction buffer were added to each well used in the assay and incubated at 37°C for 5 min. The caspase activity was measured using a caspase-3 fluorometric assay kit from Biovision. The plate was read in a CytoFluor II PerSeptive Biosystems (Farmington, MA) fluorometric plate reader with excitation at 360 nm and emission at 460 nm.

### Thiobarbituric Acid Assay

Lipid peroxidation was measured the thiobarbituric acid (TBA) assay. After 24 h of treatment of Renca cells with 90 mM Pho-s or 5 µM Sunitinib, cells were washed, counted, and resuspended in 0.2 ml PBS. To prevent lipid peroxidation 2 ml of trichloroacetic acid and 2 ml of thiobarbituric acid solution were added. The samples were heated on a thermo block for 15 min at 100°C, cooled down to room temperature, and after centrifugation at 1500 rpm for 10 min, the absorbance of the supernatant was measured in a plate reader (Thermoplate TP Reader, Tokay Hit, Japan) at 530 nm. Absorbance was converted to nmol malondialdehyde/10^6^ cells.

### Measurement of Hydrogen Peroxide (H_2_O_2_) by the Horseradish Peroxidase Assay

Renca cells (10^5^ cells/well) were grown in 6-well plates and further incubated with 90 mM Pho-s or 5 µM Sunitinib for 24 h at 37°C. Treated and untreated cells were washed three times with PBS and harvested with a cell scraper. Briefly, cells were resuspended in 1 ml of phenol red solution, containing 140 mM NaCl, 10 mM potassium phosphate buffer, pH 7.0, with 0.5 mM dextrose, 0.28 mM phenol red (PRS), and 8.5 U/ml of horseradish peroxidase for H_2_O_2_ detection. After 60 min of incubation at 37°C, the reaction was stopped by the addition of 10 ml of 1 N NaOH solution. H_2_O_2_-dependent phenol red oxidation was measured in a plate reader (Thermoplate TP Reader, Tokay Hit, Japan) at 530 nm. Absorbance was converted to nmol H_2_O_2_/10^6^ cells.

### Scanning Electron Microscopy

Renca cells were treated with 90 Mm Pho-s for 12 h, fixed with 1% osmium tetroxide (pH 7.4) at 4°C for 1 h. The samples were dehydrated, dried in a critical point drier and gold sputtered. Scanning electron microscopic (SEM) images were then taken on a LEO 453 VP, Electron Microscopy.

### Metastasis Assay *in vivo*

Renca cells (1×10^6^ cells/mouse) were injected into 6-week-old male BALB/c mice through the tail vein. The animals were randomized and divided into different groups (n = 5). On the fourth day after the tumor cells inoculation, the treatment was started with the intraperitoneal administration of Pho-s at the concentrations of 50 and 100 mg/kg/day or 10 mg/kg/day of Sunitinib and continued for 15 days. Then, the mice were euthanized and the lungs were dissected and examined histologically. The animal model experiments were carried out in accordance with the guidelines for animal experimentation determined by the Butantan Institute Animal Care committee (process number 566/09).

### Histological Analysis

Mouse metastasis tissues were first fixed with 2% formaldehyde, dehydrated and embedded in paraffin. Sections of 5 µm were obtained and stained with Hematoxylin- eosin to be screened for metastatic sites. The quantification was performed using ImageJ software freely available from the National Institutes of Health at http://rsb.info.nih.gov/ij.

### Statistical Analysis

All values were expressed as mean±SD. Each value is the mean of at least three independent experiments in each group. For significance analyses Student’s t-tests and One way analysis of variance (ANOVA) were calculated using GraphPad Prism 4.0.*p* values <0.05, 0.01 and 0.001 were considered significant.

## Results

### Cytotoxic Effects of Pho-s and Sunitinib on Renca, IRPTC and HUVEC Cells

**Figure 1:**
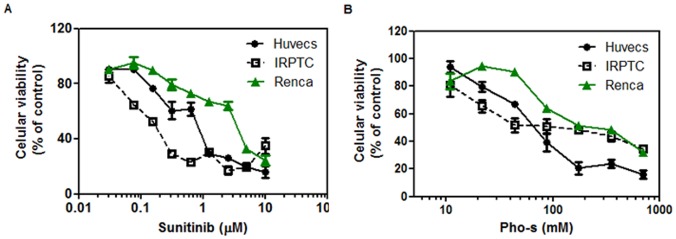
Effects of Pho-s and Sunitinib on the viability of Renca, IRPTC and HUVEC. Cells were plated at a density of 10^4^/well and treated with Pho-s (a) and Sunitinib (positive control) (b) for 24 h, and cell viability was assessed by MTT assay. Cell viability is expressed as the percentage of cells comparing the optical density (540 nm) of the treated cells with the optical density of the untreated cells. The data are representative of three independent experiments performed in triplicate.

The cytotoxic effects of Pho-s were evaluated in comparison to Sunitinib, used in this work as a positive control. Renca and IRPTC cells were incubated for 24 h with different concentrations of the compounds. The results from the MTT assay show that in all cells tested, Pho-s and Sunitinib reduce cell viability in a concentration-dependent manner. The IC50 value of Pho-s on Renca and IRPTC cells was 90 mM and 134 mM, respectively. Theses results indicate that Renca cells are more sensitive to Pho-s than IRPTC cells. At low concentrations, Sunitinib induces cytotoxic effects on Renca and IRPTC cells with the IC50 value of 5 and 0.05 µM, respectively. We also investigated the cytotoxic effects of Pho-s and Sunitinib on HUVEC, after 24 h of treatment, both compounds present cytotoxic effects on endothelial cells with IC50 values of 73 mM and 9 µM, respectively (Fig. 1A and B).

### Pho-s Inhibits HUVEC Proliferation by Arresting the Cell Cycle

**Figure 2:**
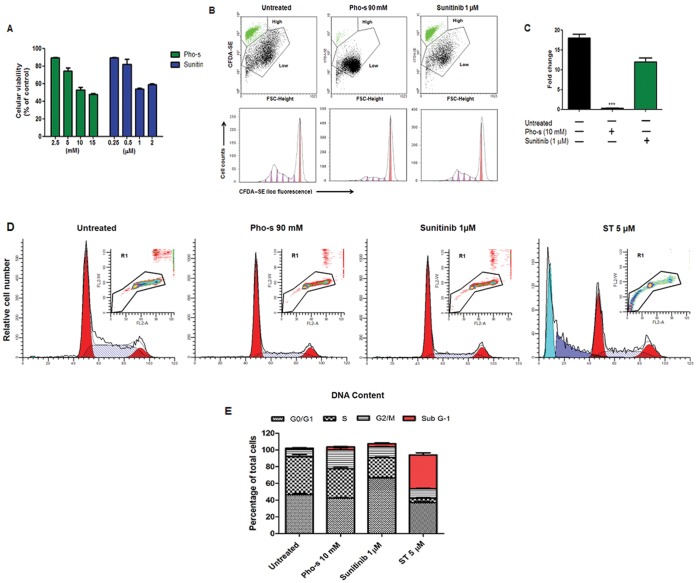
Inhibition of HUVEC proliferation. (a) Cell proliferation assessed by MTT assay shows that Pho-s and Sunitinib at subcytotoxic concentrations inhibit cell proliferation of HUVEC in a dose-dependent manner. Cells were labeled with CFDA and analysis was performed by flow cytometry. (b) Dot plot of untreated HUVEC reveals a cell population with a high division index (green). While treatment with 10 mM Pho-s shows a 4.5 fold reduction (*p<0.05) in that high division index of HUVEC, 1 µM Sunitinib did not showed a significant effects, observed after 96 h of treatment. (c) Histogram of treated and untreated HUVEC proliferation. Cell cycle analysis of HUVEC treated with Pho-s, Suninitib and Staurosporine (ST) (positive control of apoptosis) was performed by flow cytometry (d). Pho-s at the concentration of 10 mM arrests the cells at the G2/M phase (e), while Sunitinib at the concentrations of 1 µM inhibits the transition from the G1 to the S-phase and ST induces apoptosis recognized as the sub-G1- peak (e). The % of G2/M and G0/G1 arrests are shown in the bar diagram as mean ±SD from three independent experiments.

In this study, we investigated the ability of Pho-s to inhibit HUVEC proliferation at subcytotoxic concentrations. First, we evaluated the growth kinetics of the cells exposed to Pho-s as compared to Sunitinib. As shown in Fig. 2A, HUVEC were treated with different concentrations of Pho-s and Sunitinib for 96 h. Our results show that Pho-s and Sunitinib inhibit cell proliferation of HUVEC in a dose-depend manner at the subcytotoxic concentrations measured by the MTT assay. Subsequently, we also evaluated the effects of 10 mM Pho-s and 1 µM Sunitinib on HUVEC cell proliferation by flow cytometry using the fluorescent probe CFDA-SE. Notably, we observed that Pho-s was much more potent than Sunitinib, causing a 4.5 fold reduction (*p<0.05) in the high division index of HUVEC, after 96 h of treatment with 10 mM Pho-s, when compared with untreated cells. Sunitinib did not cause a significant reduction when compared to untreated cells. It shows that virtually all of the HUVEC fluorescence resided in the undivided fraction (Fig. 2B and C). Next, we performed a cell cycle analysis to investigate the anti-proliferative effects of Pho-s and Sunitinib on HUVEC growth. An analysis of the different phases of the cell cycle shows that the treatment with 10 mM Pho-s inhibits cell progression through the cell cycle by arresting them at the G2/M phase (**p<0.01) (Fig. 2D). In comparison, 1 µM Sunitinib inhibits the transition from the G1 to the S-phase, increasing the number of cells arrested at the G1 phase (**p<0.01), accompanied by a decrease of cells in the S phase (*p<0.05) (Fig. 2E and F). Additionally, we did not observe sub-diploid cells indicating the inexistence of apoptosis. In parallel, we show that ST induces apoptosis in the HUVEC, recognized as sub-G1 peak. Sunitinib and Pho-s exhibited higher potency in blocking cell progression of endothelial cells, which is consistent with the observed in cell migration and tube assays.

### Inhibition of Endothelial Cell Migration by Pho-s Treatment

**Figure 3:**
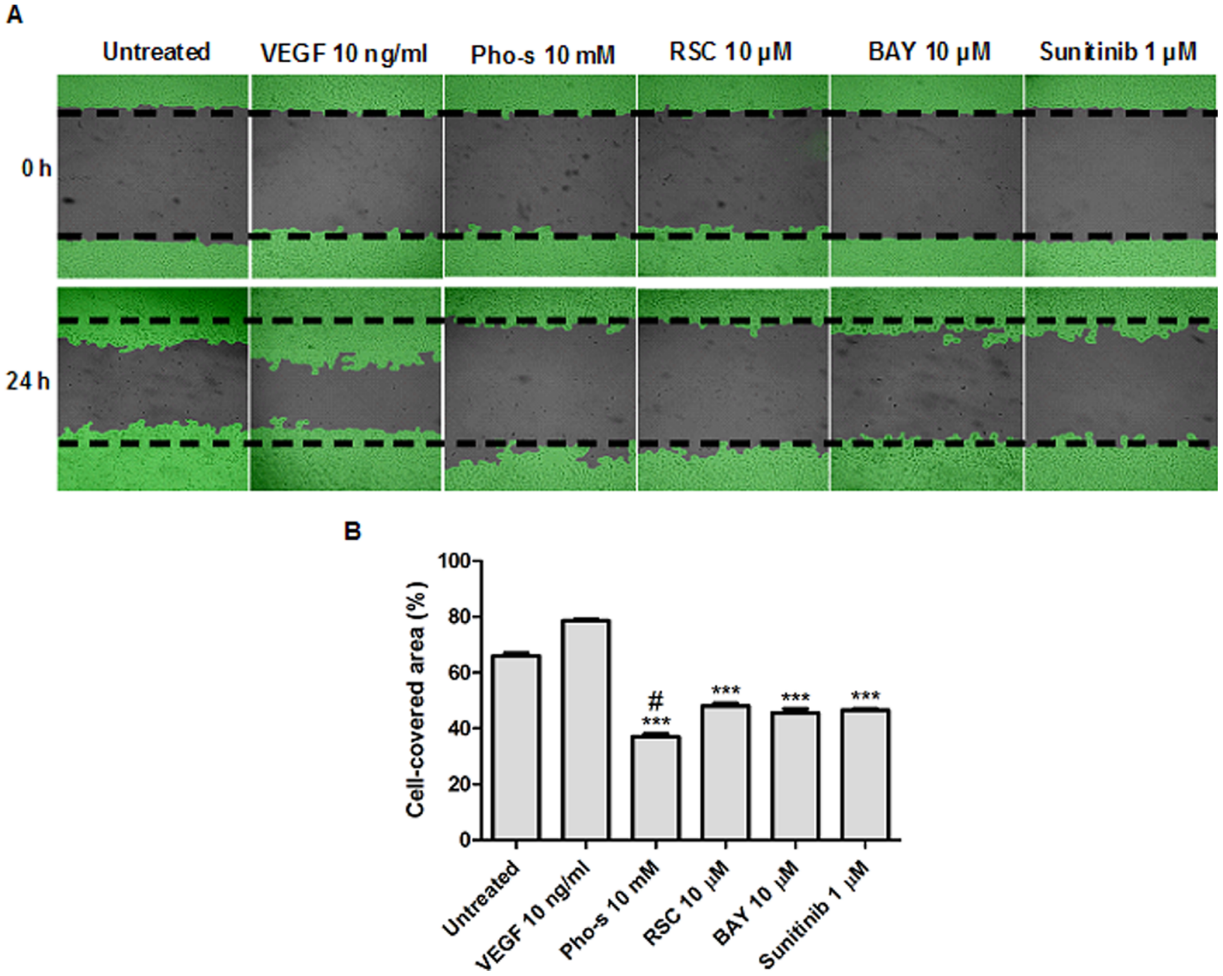
Pho-s inhibits cell migration of HUVEC as verified by wound-healing assay. (a) Representative image of the inhibition of cell migration by Pho-s after 24 h of treatment, compared to Suninitib, Roscovitine (RSC) and BAY 11-7082 (BAY). (b) The % of cell-covered area are shown in the bar diagram as mean ±SD from three independent experiments. Significant differences are indicated as: ***p<0.001 and ^#^ *p*<0.05 statistically different from the Pho-s versus untreated and versus negative controls. Images are taken immediately after scratching the cultures 0 h and 14 h later. (Original magnification, x400).

We employed an in vitro endothelial wound healing assay to evaluate the effect of Pho-s on HUVEC migration, a central process in angiogenesis during tumor growth. We compared the effect of Pho-s with Sunitinib, Roscovitine and BAY 11-7082 inhibitors (negative controls). After 24 h of treatment, we found that migration of HUVEC was significantly (***p<0.001) inhibited by 10 mM Pho-s, 1 µM Sunitinib, 10 µM Roscovitine and 10 µM BAY 11-7082 as compared to the untreated cells and those treated with 10 ng/ml VEGF. However, Pho-s was significantly (^#^p<0.05) more effective in the inhibition of endothelial cell migration in comparison with negative controls (Fig. 3A and B).

### Pho-s Inhibited HUVEC Tube Formation in 3D Culture

**Figure 4:**
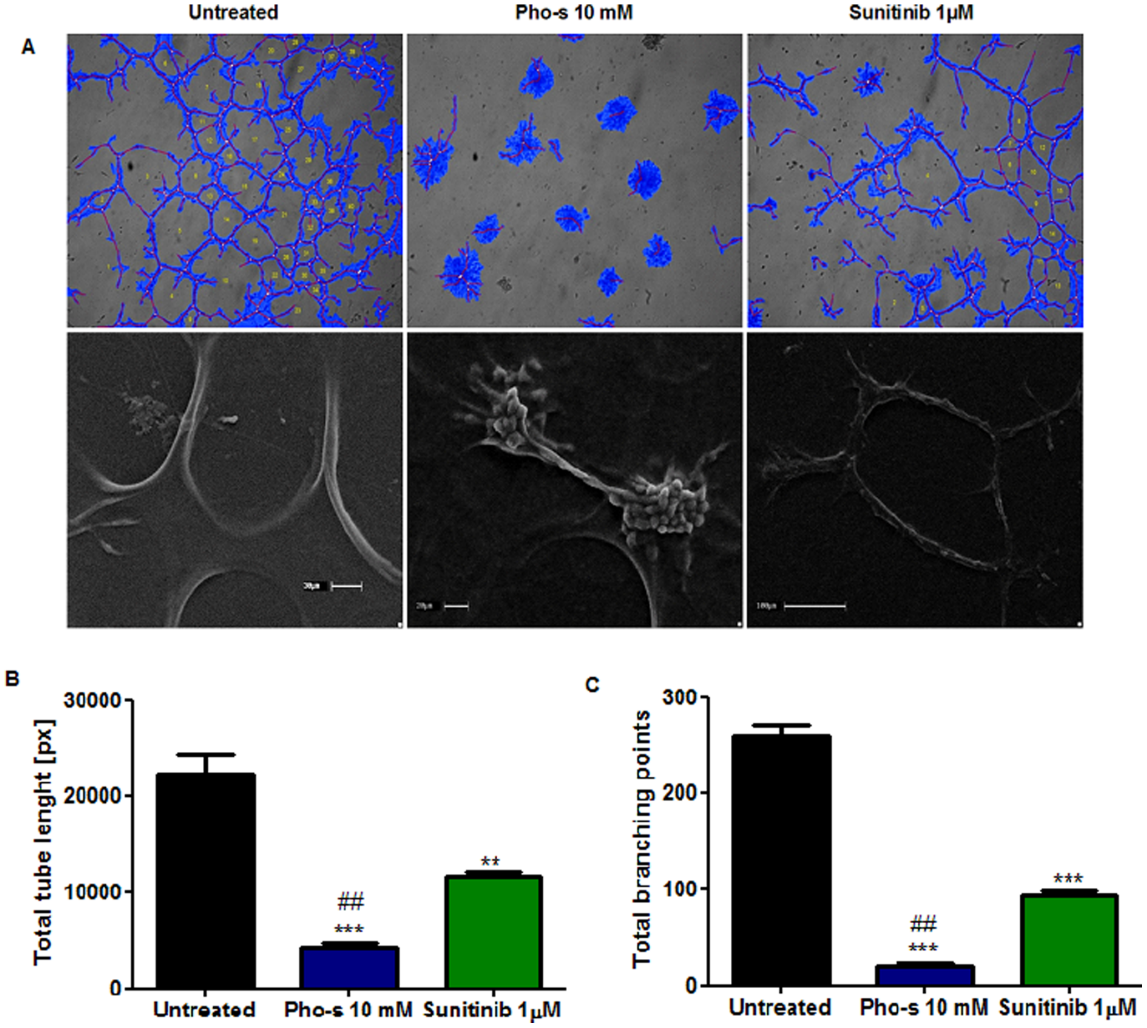
In vitro endothelial tube formation assay employing Matrigel as a three-dimensional extracellular matrix. (a) Comparative effect of Pho-s and Sunitinib in HUVEC shows that Pho-s is much potent in mediating anti-angiogenic effects, inhibiting tube formation. Of note, the cells treated with Pho-s remain adherent in spherical clusters, lacking mature tube-like structures as observed by the SEM, while Sunitinib induces morphological changes of cells correlating with inhibition of tube formation. Quantification analysis of tube length (b) and branching points (c) show that Pho-s is effective in inhibiting crucial processes involved in angiogenesis. All conditions were assessed in triplicate, and data are expressed as mean ±SD from three independent experiments.

Next, we evaluated whether Pho-s inhibits the capillary formation of HUVEC on 3D matrigel systems. Our data clearly show that Pho-s, mediated anti-angiogenic effects, inhibiting tube formation on 3D matrix (Fig. 4A). After treatment of 10 mM Pho-s, the endothelial cells did not form a network of capillary-like tubes, which were composed of multiple cells that remained in clones and adhered to each other on matrigel. In addition, the treatment with Pho-s and Sunitinib, reduces the extent of several crucial processes involved in angiogenesis, such as the area of the newly formed tube, tube length and branching points, Pho-s presenting a significantly (***p<0.001) more potent inhibition of tube formation in *in vitro* angiogenesis than Sunitinib (**p<0.01) (Fig. 4B). We further analyzed the morphological aspects of HUVEC in the tube assay after treatment with Pho-s and Sunitinib. Our findings from SEM show that endothelial cells form a capillary-like network, and are connected to each other, mimicking gap junctions, marginalizing to form the branching points. The more surprising differences between the treatments are that Sunitinib induces morphological changes on the surface of HUVEC, which can result in an inhibition of tube formation, while the treatment with Pho-s does not induce morphological changes on HUVEC. The cells treated with Pho-s were found attached to matrix gel, without evidence of membrane perturbations. Indeed, the cells change their alignment into cords, inhibiting the fusion into continuous tubes (Fig. 4C).

### Pho-s Induces Anti-proliferative and Anti-angiogenic Activity by Suppressing Cyclin D1 mRNA and VEGFR1 Gene Transcription and Receptor Expression

**Figure 5:**
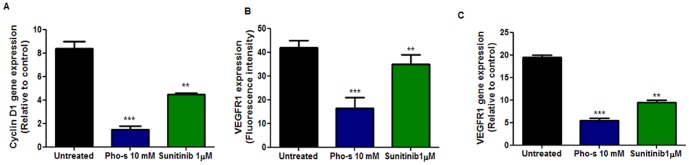
Pho-s and suninitib decrease cyclin D1 and VEGFR1 gene transcription and VEGFR1 receptor expression. (a) RT-PCR analysis of cyclin D1 demonstrated that the treatment with 10 mM Pho-s and 1 µM Suninitib reduces cyclin D1 mRNA in HUVEC. Data are expressed as fold increase versus control cells treated with PBS only and are the mean± SD of three experiments. Pho-s and Sunitinib significantly decreased VEGFR1 gene transcription (b) and (c) VEGFR1 receptor expression. The data are representative of three independent experiments performed in triplicate.

To further verify the anti-proliferative effect of 10 mM Pho-s as compared to 1 µM Sunitinib, we evaluated the cyclin D1 and VEGFR1 mRNA expression by RT-PCR of HUVEC. The concentrations of Pho-s and Sunitinib used here was the same used in the *in vitro* angiogenic assay and cell cycle analysis. Consistent with the cell cycle results, Pho-s and Sunitinib exhibit an anti-proliferative effect, decreasing the level of cyclin D1 mRNA in HUVEC after 12 h of treatment. Of note, Pho-s is much more potent (***p<0,001) than Sunitinib in suppressing cyclin D1 gene expression (Fig. 5A). In response to a variety of angiogenic stimulations, endothelial cells enter a proliferative state, and VEGF is an important inducer of this mechanism. Thus, we also evaluated whether the anti-proliferative effect of Pho-s correlates with VEGFR1 (flt1) modulation by Fac’s analysis and RT-PCR. Notably, 10 mM Pho-s causes a strong reduction of VEGFR1 expression on the surface of HUVEC, as verified by fluorescence intensity and transcriptional expression. In contrast, here in this case the treatment with 1 µM Sunitinib did not exhibit anti-angiogenic effects by modulation of VEGRR1 on HUVEC (Fig. 5B and C).

### Treatment of Pho-s Induces Disruption of Cytoskeletal Network, Formation of Apoptotic Bodies and Nuclear Fragmentation in Matrigel 3D

**Figure 6:**
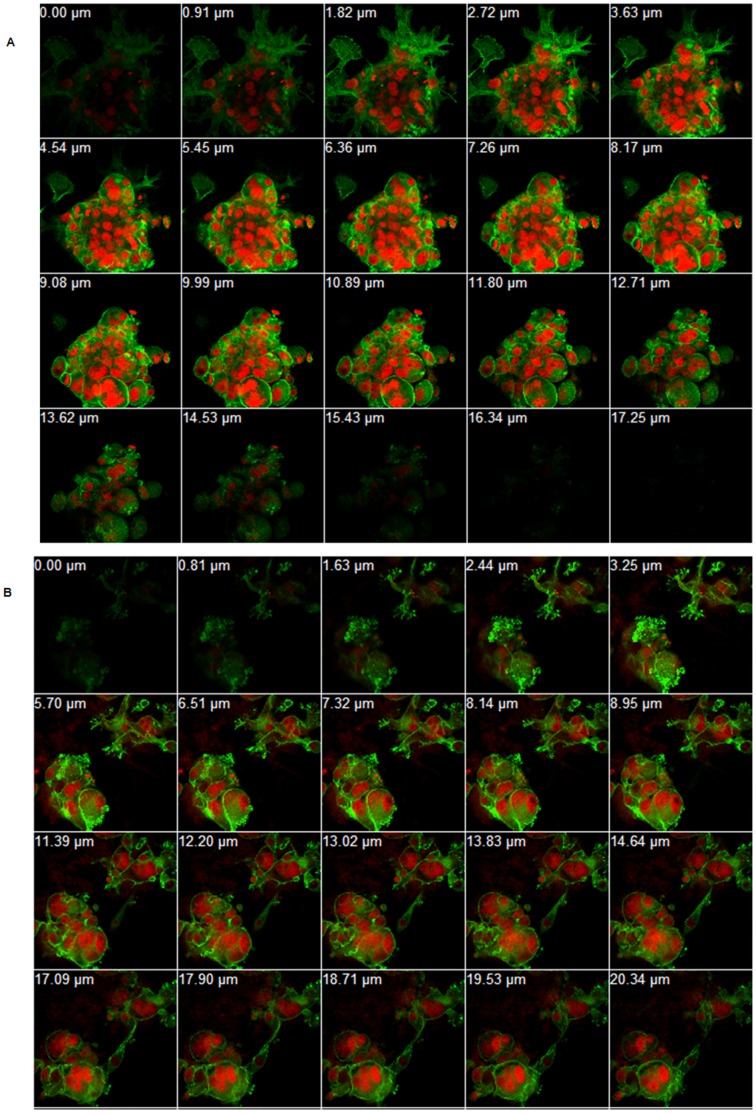
Organization of the Renca cells cytoskeleton. Immunofluorescent staining of Renca cells for the visualization of actin filaments (phalloidin, green) and nucelus (red, PI). Z-series construction of the untreated cell (a), and treated with 10 mM Pho-s for 12 h (b). Pho-s induces morphological changes in the actin cytoskeleton and induces nuclear fragmentation in Renca cells. (Original magnification x60).

In order to study the details of cytotoxic effects of Pho-s on Renca cells, cytoskeleton and nuclei were stained by phalloidin (actin) and PI (nuclei), respectively. Migration of tumor cells from the primary tumor region to the metastatic sites depends on cytoskeletal remodeling and, ultimately, on the motility of cells. Optical sections of untreated cells show a spheroid-like formation, displaying a diffuse distribution of actin throughout the cytoplasm with cells containing multiple nuclei (Fig. 6A). In contrast, the treatment of Renca cells with Pho-s induces nuclear fragmentation and irregularly distributed microfilaments in the cytoplasm, indicating a disruption in the cytoskeletal network. Furthermore, the confocal microscopy sections from cells treated with Pho-s showed brightly stained microfilaments concentrated at the site of the formation of apoptotic bodies (Fig. 6B). Collectively, these data suggest that the anti-metastatic effects of Pho-s can be associated with a marked reorganization of the cytoskeleton due to the disruption of actin and the induction of apoptosis in the Renca cells.

### Pho-s Induces the Apoptosis of Renca Cells through the Mitochondrial Pathway

**Figure 7:**
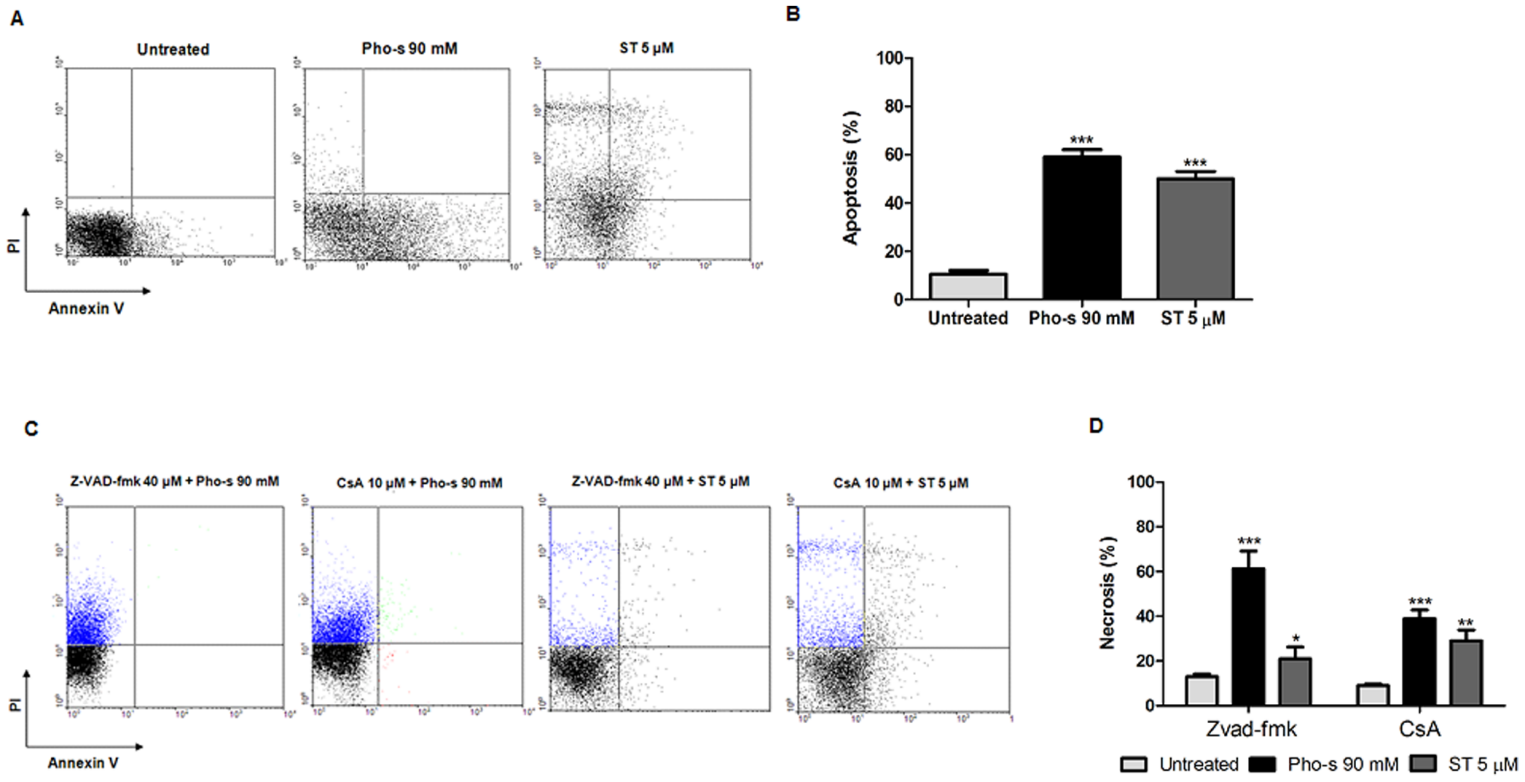
Mitochondrial membrane depolarization and caspase-3 activity induced by Pho-s in Renca cells. (a) Representative dot plots of cells stained with JC-1 dye are shown. The decrease of green fluorescence monomers correlates with the mitochondrial depolarization (Low), and cells exhibiting red fluorescence, which are viable cells (High). (b) The fluorescence intensities of JC-1 were determined by flow cytometry based on the dot plots analysis. (c) Caspase-3 activity increases were accompanied with a reduction of ΔΨ in Renca cells, induced by Pho-s and ST. The data are representative of three independent experiments performed in triplicate. Significant differences are indicated as: ***p<0.001 statistically different from the Pho-s versus untreated.

To evaluate whether the morphological changes induced by Pho-s on Renca cells correlate with apoptosis, we first demonstrated that 90 mM Pho-s induces cell death by apoptosis (***p<0.001), with the externalization of phosphatidylserine (Fig. 7A and B), a hallmark of apoptosis. To further examine the mechanism underlying Pho-s induced cell death in Renca cells, we used apoptosis inhibitors. Firstly, the role of caspases in this process was studied, and confirmed, using Z-VAD-fmk, which completely blocked Pho-s induced apoptosis. Additionally, Z-VAD-fmk reduces the potency of ST in the induction of apoptosis in Renca cells. To confirm whether the apoptotic effects of Pho-s were associated with mitochondrial permeability transition (MPT), Renca cells were treated with CsA for 2 h, prior to Pho-s and ST treatment. Our findings show that pre-treatment with CsA completely inhibits the induction of apoptosis by Pho-s. However, ST treatment showed to be partially MPT-dependent to induce apoptosis in Renca cells. Importantly, in this study we have shown that Pho-s induces (***p<0.001) necrosis in case of impairment of the mitochondrial pathway (Fig. 7C and D).

### Pho-s Induces Mitochondrial Depolarization and Increases Caspase-3 Activity in Renca Cells

**Figure 8:**
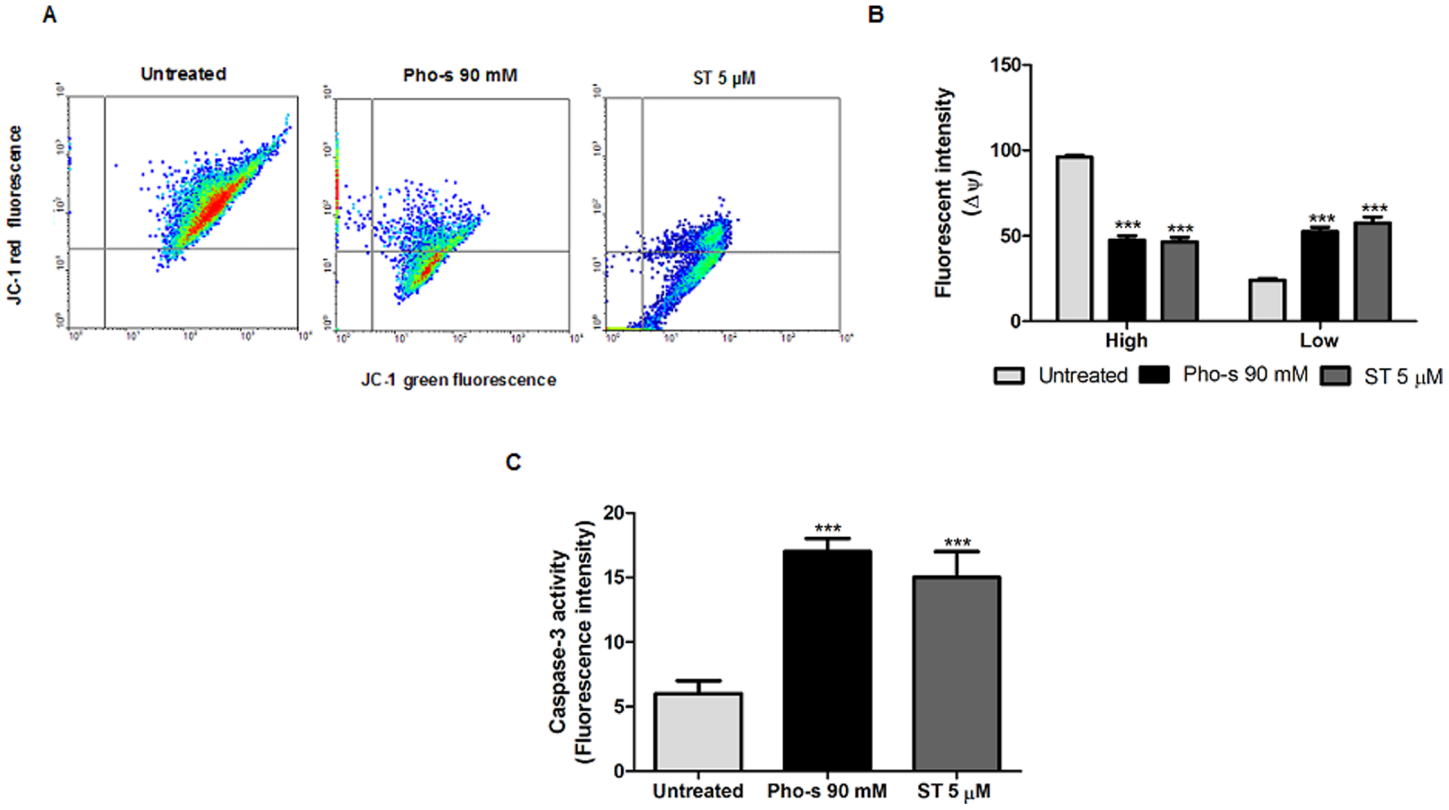
Pho-s induces apoptosis though the mitochondrial-dependent pathway in Renca cells. (a) Dot plot profile represents V-FITC/PI staining showing the percentage of apoptotic cells. (b) An increase of apoptotic cells was observed after 12 h of treatment with 90 mM Pho-s. (c) The mitochondrial dependent mechanism was evaluated following pretreatment with 40 µM Z-VAD-fmk or 2.5 mM cyclosporin A (CsA), and evaluated by V-FITC/PI staining using flow cytometry. (d) The increase of necrosis induced by Pho-s and ST indicated that Pho-s induces its apoptotic effects through the mitochondrial-dependent pathway. The data are representative of three independent experiments performed in triplicate.

Having found that Pho-s induces apoptosis in a mitochondrial-dependent pathway, the alteration on mitochondrial function was investigated. We measured the mitochondrial transmembrane potential (ΔΨ) by staining with 10 nM TMRE after treatment with 90 mM Pho-s or 10 µM ST. As shown in Fig. 8A, Pho-s and ST induced mitochondrial depolarization as indicated by a decrease in the red to green fluorescence intensity ratio in Renca cells. From these results, it became clear that both Pho-s and ST reduce ΔΨ as shown by the cell population in low metabolism mitochondrial as indicated by fluorescent intensity (***p<0.001) (Fig. 8B). Accordingly, the reduction of ΔΨ facilitated cytochrome c release from the mitochondria to the cytoplasm, which in turn induces caspase activation. Consistent with the ΔΨ reduction, we demonstrated that Pho-s and ST induces a marked significantly increase (***p<0.001) in the activity of caspase-3 (Fig. 8C). Theses results corroborate the above results showing that Pho-s-induced apoptosis is mediated by a mitochondria-dependent pathway.

### Pho-s Induces Changes in the Oxidative Status on Renca Cells

**Figure 9:**
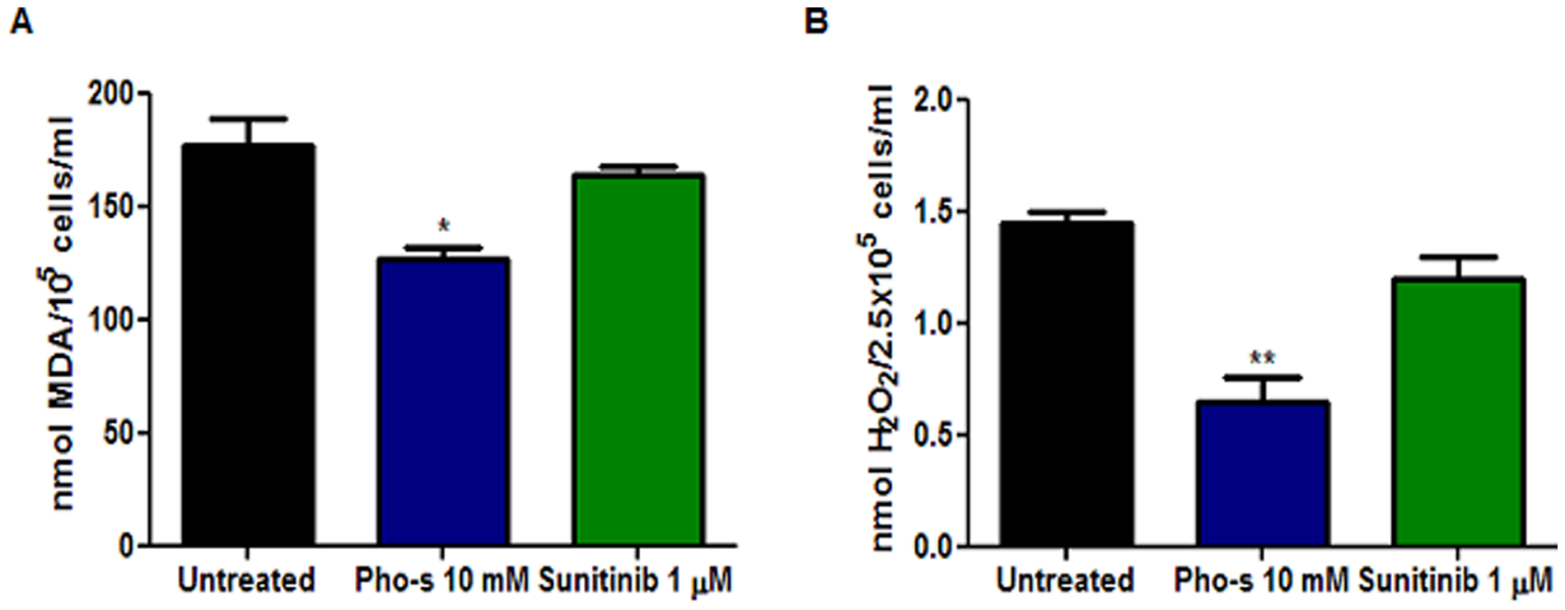
Lipid peroxidation and hydrogen peroxide formation. Renca cells were treated with 90 mM of Pho-s or 5 µM Sunitinib for 12 h, and then were analyzed as described in Materials and Methods. The detection of MDA and H_2_O_2_ was evaluated through the thiobarbituric acid and horseradish peroxidase assay, respectively. The data are representative of three independent experiments performed in triplicate.

We also evaluated whether the anti-angiogenic effects of Pho-s correlate with a change in the status of oxidative stress. We first measured the index of lipid peroxidation through the formation of malondialdehyde (MDA) and other low molecular weight aldehydes, which by reacting with 2-thiobarbituric acid (TBA) form Schiff bases. Our results show that 90 mM Pho-s significantly (*p<0.05) reduces the levels of malondialdehyde as compared to the untreated cells and those treated with 5 µM Sunitinib (Fig. 9A). The production of mitochondrial reactive oxygen species by Renca cells was estimated by detection of hydrogen peroxide, using a classical horseradish peroxidase method. Concomitantly, the results show that hydrogen peroxide (H_2_O_2_) formation was also significantly reduced (**p<0.01), indicating that Pho-s has protective effects which are reflected in a reduction of oxidative species. Theses results demonstrate that Pho-s changes the oxidative stress status of Renca cells, and anticipated the *in vivo* therapeutic outcome (Fig. 9B).

### Pho-s Inhibits *in vivo* Lung Metastasis Colonization of Renca Cells in BALB/c Mice

**Figure 10:**
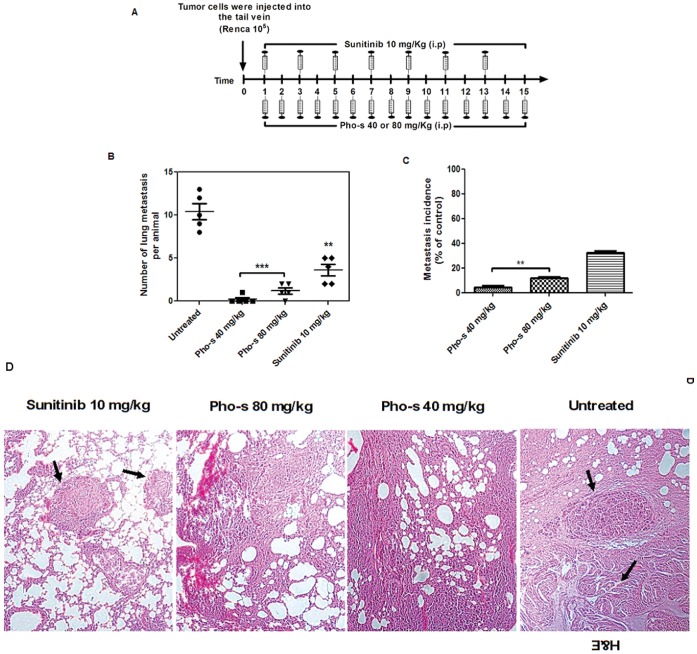
Pho-s inhibits lung metastasis of Renca cells in BALB/c mice. (a) Schedule of treatment of Pho-s and Sunitinib. 1×10^6^ Renca cells were injected via the tail vein, and on the fourth day after the tumor implant, the treatment (i.p.) was started with Pho-s at the concentrations of 50 and 100 mg/kg/day and 10 mg/kg/day of Sunitinib and continued for 15 days. Both concentrations of Pho-s, significantly (***p<0.001) reduced both the number of lung metastasis per animal (b) and the incidence of metastasis (c), when compared to untreated mice and to treatment with Sunitinib. (d) H&E sections of lung metastasis 15 days after tumor inoculation from untreated mice and mice treated with 10 mg/kg Suntinib show the multiple metastatic foci (arrows), while in mice treated with Pho-s at both doses almost no metastatic foci can be seen. (Original magnification x20).

We injected BALB/c mice with Renca cells via the tail vein and observed tumor formation in the lungs 2 weeks after injection. The pulmonary metastatic number of the treated mice versus untreated was assessed by counting the number of tumor nodules on the surface of the lung on the 15^th^ day of treatment (Fig. 10A). All untreated animals (100%) had multiple metastatic nodules in the lung with opaque spots characterizing tumors, and several had tumors in other organs. The most interesting finding was that the treatment with Sunitinib and Pho-s reduced not only the number of metastasis in the lung, but also their dissemination to other organs of the body (total nodules). Additionally, the macroscopic analysis confirmed that Sunitinib (**p<0.01) reduces the number of lung metastatic foci. Furthermore, the treatment with 40 mg/kg/day of Pho-s completely inhibits the appearance of metastatic foci in the lung, while the treatment with 80 mg/kg/day of Pho-s decreased the number of lung metastatic nodules than untreated mice and Sunitinib treatment (***p<0.001) (Fig. 10B and C). A histological examination was performed using H&E staining to identity colonies in the lungs of mice that had received Renca cells intravenously. It is interesting to note that tumor sections of untreated mice are in agreement with macroscopic findings. A noticeable increase in metastatic colony density and in the number of cells tumor with mitotic nuclei was observed. In contrast, the histological analysis conform the significantly suppressed cancer metastasis to the lung by Pho-s. Almost no metastatic foci could be detected in these tissues from mice treated with Pho-s at both doses, while in the lung of mice treated with Sunitinib, clearly more metastatic foci were seen (Fig. 10D). Thus, these *in vivo* findings provide support for the conclusion that Pho-s has high anti-metastatic properties.

## Discussion

It has been reported that Pho-s has potent antitumor effects *in vitro* against a variety of tumor cells. In a melanoma model study, the treatment with Pho-s inhibited tumor progression and increased animal survival rate. An additional highlight of this study is the finding that around the dorsal tumor of mice treated with Pho-s no neovascular region could be seen [18]. This raises the question of whether the antitumor mechanism of Pho-s is mediated by its anti-angiogenic activity. Here we show, for the first time, the anti-metastatic effects of Pho-s on Renca cells and *in vivo* animal model. Although Pho-s and Sunitinib induced significant cytotoxic effects in all the tumor and normal cell lines used in this work, we show that Renca cells are more sensitive than HTRP and HUVEC to Pho-s, while Sunitinib is more cytotoxic to endothelial cells. This is also in agreement with our earlier observations showing that Pho-s is more selective to tumor cells than to normal cells [19]. Thus, these findings further support the *in vivo* results showing that Pho-s can be used at therapeutic concentrations without or lower side effects.

The present study was designed to evaluate the anti-angiogenic properties of Pho-s. Our first step was to investigate if Pho-s can regulate two key steps in the angiogenic process: endothelial cell proliferation and migration. One unanticipated finding was that Pho-s, at a subtoxic concentration, has an anti-proliferative activity, inhibiting HUVEC growth. In accordance, we have demonstrated, using the CFDA-SE probe, that Pho-s is clearly more potent than Sunitinib in the inhibition of cell proliferation, reducing the division index of HUVEC.

Endothelial cell migration is a crucial step of angiogenesis, and is regulated by many stimuli that induce degradation of the extracellular matrix, which enables progression of the migrating cells to form new blood vessels [20], [21]. In this study, we demonstrate that Pho-s, at a low concentration, inhibits the migration of HUVEC as effectively as Sunitinib (multikinase inhibitors), Roscovitine (selective inhibitor of cyclin-dependent kinases) and BAY 11-7082 (inhibitor of κB kinase). While this preliminary finding suggests that Pho-s has potent effects when compared to other inhibitors, it is not possible to infer that its mechanism of action is the same. The inhibition of cell proliferation can be associated with perturbations of the cell cycle. Thus, we sought to determine whether Pho-s blocks HUVEC cycle progression. In the current study we found that Pho-s can inhibit HUVEC proliferation by arresting them at the G2/M phase.

Our data clearly show that the anti-proliferative effects of Sunitinib occur through the blockage at the G1/S transition and the increase in number of cells in G1 was clearly related to the decrease of cells in the S phase. Interestingly, we did not observe any events preceding the detection of apoptotic cells recognized as sub-diploid “Sub-G1” peak, as opposed to the effects of the treatment with ST. Since we showed that Pho-s inhibits cell cycle progression we next investigated whether cyclin-D1 might be mechanistically involved in the perturbation of cell cycle. It has been reported in previous studies that down regulation of the cyclin D1 mRNA reduces proliferation rates and induces cell cycle arrest without affecting cellular viability. Interestingly, both Pho-s and Sunitinib down regulate cyclin D1 mRNA, but Pho-s is more effective than Sunitinib in reducing the expression of the gene. In contrast to Pho-s, the degree of cyclin D1 mRNA down regulation in HUVEC treated with Sunitinib did not clearly correlate to the arrest in G1. These observations suggest that down regulation of cyclin D1 mRNA and the subsequent cell cycle arrest at G2/M induced by Pho-s may be due to a cellular response to the loss of signals from the extra cellular matrix [22].

To complement our results showing that Pho-s inhibits endothelial cells proliferation and migration, we also demonstrate that Pho-s affects the capillary network formation *in vitro* at a sub-cytotoxic concentration, inducing a decrease of the total capillary length and number of branching points, which are known to be crucial for angiogenesis [23], [24]. To further evaluate the capillary network formation, we employed SEM to observe details of the mesh. We verified that Pho-s does not affect the overall morphology of HUVECs on a 3D matrix, which form a well-organized capillary-like structure similar to the untreated cells. On the other hand, Sunitinib induced modifications of the cell morphology, even at a low concentration.

Anti-angiogenesis therapy with focus on VEGF receptor inhibition is a promising strategy for targeting solid tumor growth. It has been widely reported that VEGF is a key factor in the development of tumor angiogenesis by stimulating endothelial cell proliferation, migration, and capillary tube formation [25], [26], [27]. In this study, we found that VEGFR1 expression, as shown by flow cytometry, decreases in Renca cells upon treatment with either Pho-s or Sunitinib. Moreover, Pho-s was more potent in reducing VEGFR1 than Sunitinib. Further analysis of the VEGFR1 expression profile in HUVEC was carried out by RT-PCR, which confirmed the flow cytometry data. We demonstrate a down-regulation of VEGFR1 mRNA by Pho-s, evidencing a regulation at the transcriptional level. Although the exact mechanism of action through which Pho-s modulates VEGFR1 in HUVEC is still under investigation, our results show that Pho-s down regulates VEGFR1, at least at the transcriptional level. Its inhibition by Pho-s would reduce the effects of VEGF and other specific ligands, which suggests a mechanism for Pho-s induced inhibition of metastasis. Of note, the cyclin mRNA and VEGFR1 down-regulated by Pho-s are most likely associated with a dramatic inhibition of cell migration, proliferation and capillary tube formation.

A precondition for tumor cell migration and metastasis is a functional and dynamic cytoskeleton, in order to produce the necessary protrusions and forces that drive the cell to other tissues [28], [29]. The cytoskeleton also plays an important role in the processes of growth and cell differentiation and is involved in the signaling pathways downstream of receptors that lead to the remodeling of the actin filaments [30]. It is crucial for the motility and chemotaxis of tumor cells, which in turn influence the metastatic ability of tumor cells [31]. Importantly, Pho-s affects the organization of the cytoskeleton, which could in turn affect the cellular organization, adhesion complexes, cell polarity and vesicular transport. Pho-s might able to inhibit tumor metastasis and cancer progression through the disruption of actin filaments of Renca cells.

We then investigated whether the disruption of the cytoskeleton is a sufficient condition to trigger the apoptosis of Renca cells. In fact, cytoskeleton disruption induced cell death by apoptosis, detected through phosphatidylserine externalization. Based on these data, it is reasonable to suggest that these morphological changes were mediated by a cysteine protease, which is in agreement with the increased caspase-3 activity. It suggests that Pho-s may induce apoptosis via the intrinsic pathway. It is also interesting to notice that, as opposed to its effects on Renca cells, Pho-s did not induce apoptosis and necrosis of HUVEC (date no shown). In our study, the decrease of ΔΨ in Renca cells in response to Pho-s is an evidence of mitochondrial dysfunction, an early event in the process of apoptosis [32]. Interestingly, using CsA and Z-VAD-fmk, we confirmed that Pho-s induces apoptosis through the mitochondrial-dependent pathway. This assumption is based on the fact that Pho-s causes necrosis when caspases and MPT are blocked. In the present study we show that the pro-apoptotic activity of Pho-s is similar to that of ST. Our results show that Pho-s can achieve the same apoptotic effects as ST in Renca cells.

Metastasis is the major cause of death in cancer patients. Tumor growth and invasion are dependent on angiogenesis and on its interaction with extracellular matrix in order to spread into the surrounding tissue. It involves a multi-step process, allowing tumor cells detachment from the primary tumor site and migration to secondary sites [33]. Here, we provide *in vitro* evidence that Pho-s inhibits tumor metastasis and that it can be associated with the inhibition of angiogenesis as well as with the inhibition of tumor cell migration. However, we still had to verify *in vivo* whether Pho-s effectively inhibits the development of metastasis. To accomplish this goal, we used a highly aggressive model of murine renal carcinoma metastasis and compared Pho-s with Sunitinib, a standard drug for the treatment of patients with advanced RCC that has been approved by the Food and Drug Administration. In this preclinical study, the effects of the *in vivo* administration of Pho-s were compared to Sunitinib, as detailed in materials and methods. Both procedures inhibited lung metastasis in comparison to untreated mice; moreover, the antitumor efficacy of Pho-s was found to be much higher than that of Sunitinib. We also demonstrated that the treatment with Pho-s at all concentrations was well tolerated and it significantly reduced the frequency of lung metastasis. Interestingly, our remarkable *in vitro* results show that Pho-s decreases MDA and H_2_0_2_ oxidative stress. Both MDA and H_2_O_2_ act as second messengers, and have been shown to be important mediators involved in tumor metastasis. Of note, curative resection of RCC does not reduce oxidative stress, which is frequently increased in patients with metastasis and residual disease [34], [35]. It has been extensively demonstrated that the production of ROS play a critical role in angiogenesis, invasion and metastasis. We could hypothesize that the decreased oxidative stress is associated with the anti-metastatic activity of Pho-s. Thus, the reduction of oxidative stress induced by Pho-s could reduce tumor growth, metastasis, and angiogenesis. Additionally, a reduction in ROS in response to Pho-s can decrease HIF-1 expression, whose inhibition is known to reduce tumor progression [36]. Further studies should be done to investigate this hypothesis.

Notably, the reduction of ROS prevents additional damages to the DNA of tumor cells, reducing cell proliferation [37]. These findings were confirmed by the histological analysis. In untreated mice many metastasis foci were observed with increasing numbers and frequency of tumor nodules. In contrast, Pho-s inhibited lung metastasis, with very few foci observed, while Sunitinib presented an advantage relative to untreated mice, but a moderate effect when compared to Pho-s at both concentrations.

In summary, we found that Pho-s has an *in vitro* anti-angiogenic activity, inhibiting important and essential steps in the angiogenesis process, such as cell proliferation, migration and the formation of capillary-like tubes. We demonstrate here that Pho-s can inhibit metastasis through the disruption of actin filaments, which in turn can prevent tumor cell migration. Indeed, Pho-s induces apoptosis through a mitochondria-dependent pathway in Renca cells. Altogether, our *in vivo* study strongly supports the potential of Pho-s as an anti-metastatic agent, with its superior effects in comparison to Sunitinib in inhibiting lung tumor colonization. Overall, Pho-s is a compound that potently inhibits metastasis, suggesting that it is a promising novel drug for future developments.
